# Preventive Medicine

**Published:** 1875-07

**Authors:** Charles C. F. Gay

**Affiliations:** Buffalo, N. Y.


BUFFALO

### Medical and Surgical Journal.

VOL XIV. JULY, 1875. No. 12.

### Original Communications.

:o:

ART. 1.—*Preventive Medicine.* An address delivered June 8th, 1875, before the Medical Society of Erie county. By CHARLES C. F. GAY, M. D., Buffalo, N. Y.

It was Headland, an English physician, who said that the object of the art of healing is to prevent man from dying before his time.

According to the Scriptures, the time for man to die, is when he has attained to the age of three score years and ten, but if perchance, by reason of strength he arrive in his earthly pilgrimage to four score years it is man's misfortune, since sorrow, with mental and physical infirmity, is indissolubly connected with that advanced period of life.

Two ways there are of preventing man from dying before his time. One way is to prevent illness. Another way is to cause the sick man to recover from an illness. Hence the division of medicine into *preventive medicine* and *curative medicine.* The former, much more neglected than the latter, is by far the more important of the two.

It is a trite but true saying, that an ounce of preventive is better than a pound of cure.

Natural history of disease has wrought a radical change in our *methodus medendi.* If the duration of disease hath a self-limitation, if there be certain specific laws which must be obeyed in disease as well as in health, and if we come to learn by observation that any well defined disease tends to terminate in recovery after having run for ten, twenty or thirty days, the physician, having knowledge of these facts, will wisely abstain from attempts to cure since he can only hope to guide his patient or the disease of his patient like a skillful mariner his vessel, during a storm, on the summit of the waves, and bring it safely into port. Although little medicine may be required the services of the physician are just as requisite. Skill does not any more consist in a knowledge of what medicine to administer than in a knowledge of when to discontinue or withhold a medicine. A correct diagnosis, and a knowledge of the natural limitation of the disease being given, the remedy will suggest itself, and more often then otherwise it will be found that the true physician differs from the false in that, while the latter administers his thirtieth dilutions, the former will give nothing but advice. But it is not my purpose to occupy your time in discussing that branch of medicine denominated curative, rather let me direct attention to the more important branch denominated preventive medicine.

Preventive medicine is a term employed to denote compliance with the laws of hygiene, it includes, and indeed is nearly synonymous with the term hygiene. It embraces a knowledge of the causes of disease, it teaches how to avoid disease, and hence assumes the position that many diseases or their causes are preventable, and that by knowledge rightly applied they may be avoided.

It reasserts the doctrine, that the people perish from want of knowledge.

The child from want of knowledge thrusts its fingers into the flame. For want of knowledge the adult exposes himself to an atmosphere pregnant with mephitic gases, and violates in various ways the laws of his being, and attributes the necessary payment of the penalty to the mysterious ways of Providence.

"Natural calamities," says Coleridge, "that do indeed spread devastation wide, (for instance the Marsh Fever,) are almost without exception voices of nature in her all-intelligible language—do this; or cease to do that." "By the mere absence," says the same writer,

"of one superstition and of the sloth engendered by it, the plague would cease to exist throughout Asia and Africa."

There are still plague spots upon the earth, and if the plague is ever stopped pilgrimages to Mecca must cease.

In his great speech made at the alumni dinner last February, Dr. Moore said, if I rightly understood him, that no advance had been made in, or no new fact added to sanitary science for the past two thousand years.

This is a startling assertion, and as a general statement or proposition, may perhaps not be discredited. The learned doctor's mind was probably running back to an earlier period in the history of the world, at a time when Jewish laws were in vogue and before their decadence, when the Jewish government was indeed a government by Divine right, with a code of laws dictated by God.

The diminution of the death rate and the average present duration of human life, may be cited to disprove the non-advance of hygiene. According to the statistics of Dr. Jarvis, the length of life has increased from an average of thirty years under the Roman empire, and twenty-one in the sixteenth century to fifty years at the present time. The unselfish labors in the domain of hygiene and devotion of a profession to prevent what seemed only to be its province to remedy, has been its distinguishing characteristic and true glory, and for these labors it is entitled to the good opinion of the world.

Preventive medicine has not been successful in eradicating disease, nor can it hope to be, but in comparison with the other learned professions the glory of the medical profession cannot be dimmed or turned to shame, since it has done as much to eradicate disease as law has done to eradicate crime or theology sin.

Sacred and secular history have been carelessly read if the fact has escaped observation that sanitary laws were highly regarded more than three thousand years ago. Indeed, in the time of Moses and the Prophets, and in the days of the glory and renown of Greece and Rome, whilst under their highest state of cultivation in literature and the arts, sanitary laws were enacted, enforced and homage paid them by that ancient people and by those ancient nations.

The system of drainage and sewerage of Rome in her palmiest days, was equal to, if not superior to any system of more modern times. Yet, if it be true, as it seems to be conceded, that preventive medicine has not made that advance during the past centuries that human health and longevity demands—that the goddess of health has really aborted, and that the cause lies in the reason given by the distinguished doctor before alluded to, namely, that the laborer is worthy of his hire; then it behooves the State and National authorities to make liberal appropriations in behalf of the advance of preventive medicine to the end, that it may be pushed forward to its completest fruition, to discover if there be "no balm in Gilead, and to know the reason why the health of the daughter of my people, is not recovered."

It would be idle to attempt to maintain the position that all diseases are preventable by the application of all the wisdom derived from a profound knowledge of the laws of hygiene. Disease would seem to be what I declared it in an address delivered many years since, before the Medical Association of this city, namely—the *normal* condition, while health is the *abnormal* condition of our race. It cannot be argued that the primeval curse has aught to do with this normal or abnormal condition of man. God not only created man in his own image, but for the good of man created He the vegetable and the mineral kingdoms, and giving to the lower order of animals, if not intelligence and reasoning faculties, at least instinct. God in the creation of the plants made them very beautiful and useful in their kind, giving to many of them medicinal properties peculiarly adapted to the ills to which "flesh is heir." Medicinal plants are coeval and co-existent with man. It would appear that disease was fore-ordained, and medicines made to grow upon or in the earth in order to combat disease by their medicinal virtues. There are animals, that when sick, possess the instinct to chose the plant which is adapted to their cure. The cinchona tree grows most luxuriantly in those marshy districts of country, which give rise to malarial diseases, such as intermittent and remittent fever, so wise and thoughtful has been the Creator to provide for the necessities of all His creatures. We cannot hope to eradicate disease by our perfection of sanitary laws since disease is the normal condition of our race, still disease or its causes may in a large measure be prevented, and will be prevented, the severity of disease mitigated and its duration abridged by just such methods as are now being put into requisition in this State, and in the United States, namely, the enlistment of the best medical talent of our country to the subject of hygiene. Preventive medicine is to become the all-engrossing subject, and a better understanding of the causes of disease is to be sought for. The intimate relation between causation and treatment, render it an imperative necessity to carry investigations further than they have been carried heretofore; indeed the intimate relationship subsisting between causation and therapeutics, is such that if we are able to have positive knowledge of the cause of any individual ailment, as for instance a remittent fever, we shall find that prolonged use of medicines will not be required, but instead of medication, removal of the patient from the cause is all that is necessary. If we cannot remove the cause which has produced an illness, and which most persistently in spite of remedies, prolongs an illness into days, weeks, or even months, recourse may be had to the other horn of the dilemma, and the patient removed away from the cause of illness to some locality where recovery will follow as a natural sequence without the aid of medicine, but with the aid of air that is salubrious and unimpregnated with malarial germs.

Our Materia Medica does not abound with prophylactics. Medicines are, in their nature for the most part curative agents, they are sought after for their curative and not for their prophylactic properties, still our works on Materia Medica furnish articles which enjoy a good reputation as preventive; thus, quinine is not only a curative agent but a preventive one as well. A suitable dose of this alkaloid will prevent the initial chill which preceeds and ushers in the fever.

The efficacy of cinchona bark in preventing and curing disease, and the vast benefit it has conferred upon mankind has never yet been estimated. Had its discovery been made a quarter of a century earlier, or had James the VI of Scotland been born a quarter of a century later he would have accomplished an enduring work for the christian world, and would not have died prematurely of a tertian ague. Cinchona bark early became popular in England, and its use advocated by the illustrious Sydenham, but too late to save the life of Cromwell, who died a few years before of an ague which it is said, the physicians could not cure. If it had been used a few years earlier the life of the Protector would have been saved and the history of England re-written. Such are examples of the influence of a single drug exerted upon the fate and fortune of nations and individuals.

Preventive medicine has not its highest sphere of usefulness as represented in the protective potency of drugs. It has a far wider range and broader scope than this. Man in his highest physiological state possesses an organic system so delicate as to be very sensitive and responsive to the influence of toxic elements when attacking either from within or without. Health and life are overwhelmed at once by the sudden onslaught of poisonous agencies. But man despite his delicate organization possesses wonderful tolerance against the gradual introduction of poisonous agents into the system. Habitual opium eaters will tolerate thirty grains of morphia daily; arsenic may be taken with impunity in doses that would otherwise excite vomiting or perchance destroy life, had not the system become gradually accustomed to its use. A bad atmosphere or mephitic gases will seem to be resisted for a time by the healthy, but like poisons taken into the stomach will *tell* upon the organism in time. The Nemesis of destruction will come and the penalty will sooner or later have to be provided for.

The writer has known a person to take six or eight different kinds of medicine daily, one of which was a mineral, certainly not of the highest "trituration," for the space of from four to six weeks, and is yet alive, indeed was not made sick, but was salivated. According to the medical gospel of those who are faithful to one idea, Russian baths or perpetual residence at Clifton, is all that is requisite and necessary, in order to eliminate from the system what has in this case been got into it.

Temperance and moderation in eating and drinking should be early considered in treating upon the subject of this paper. I state upon the authority of Dr. Brunck, of this city, that more people are injured by intemperance in eating than by intemperance in drinking.

One need not become a glutton before he becomes a dyspeptic. One may become a dyspeptic by the moderate consumption of dainties, and the commingling of substances in the stomach, such as the culinary art furnishes. Eating at unseasonable hours is pernicious. Some persons, with a smattering of physiological knowledge, argue themselves into the belief, that their brains work more smoothly and easily by abstinence from eating until their mental labor is over for the day. This is one of the follies of a false philosophy and a "little learning," Men and women of sense ought to eat an early breakfast, and dine soon after midday, and then it would not make much difference whether or not they ate again until the next breakfast time, provided they do not impair their gastronomic powers by indulgence in eating late suppers. The laws of health are as surely violated in what we eat, as in what we drink. Immoderate use of rum, whiskey, tea, coffee, and beef are alike, in degree, injurious. I have known persons to be as immoderately addicted to, and nervously injured by the use of strong tea and tobacco, as I have known other persons to be addicted to, and injured by the use of alcoholic drinks.

One of the anomolies of our age and race, as well also, inconsistencies, is the habit of some, whose prejudices will only permit them to swallow a sugar-coated pellet, while they daily consume, on an average, from one to two ounces of tobacco.

Will not some goodly apostle of temperance just turn his attention for a season to intemperance in eating.

The larger field for the exercise of sanitary tactics is found in the surroundings of our abodes and their defective construction. Good air, water and ventilation are necessary to good health. Poor air, water and ventilation are furnished in abundance, and are causes of disease and death. Houses built with all the "modern inprovements," not unfrequently become laboratories for the manufacture of impure air and poisonous gases. Modern improvements are very comfortable and agreeable in their way, but they do not tend to make healthy and vigorous men and women out of sickly and puny children. Children will continue to be sickly until these so-called modern improvements are improved upon by careful revision.

Children reared under the advantages, or rather disadvantages, of modern architectural improvements, cannot hope to grow up to strength and vigor, but will remain feeble, like a house plant. It is pleasant to be born with a silver spoon in one's mouth, and to be reared in the lap of luxury and ease; it is pleasant also, and agreeable to witness marvels of neatness, tidiness and cleanliness, for they are akin to godliness, but it has often and truly been said, that dirt is not altogether unwholesome. Bringing a child up in the way he should go, according to the wishes and predilections of the parents, is not always the best way.

The greatest hindrance to success in life of many, who are to the manor born, arises from the follies, if not the vices, of those who have begotten them; by which a feeble race is perpetuated, and a strong one enfeebled.

The open fire-place or grate should never be neglected by any one, who builds a house. The air of an otherwise close room will become decarbonized and deodorized by the fire in the open grate, and by the interchange of currents of air, which the open grate causes.

In the olden times, before stoves and furnaces came in vogue, when the back-log was in position in the open fire-place, typhoid fever was a disease comparatively unknown.

Those were days when asthenic disease could not find lodgment in the human economy, because of the invigorating influence imparted by an atmosphere purified by fire.

The open grate, whether there be fire on the hearth or not, is one of the foremost agencies in facilitating convalescence of the sick, of preventing disease, of mitigating its severity, and of abridging its duration.

The healthful tendencies of the grate fire will measurably counteract and neutralize the unhealthful tendencies of the air furnace, while the latter will keep one warm, the former will keep one healthy, therefore the simultaneous employment of both is desirable whenever and wherever the furnace cannot be dispensed with.

"A city as an unwholesome fact" is the rather curious caption of an able article written for and printed in the Sunday edition of the Courier of last February, the author of which is Joseph N. Larned, Esq., of this city. It considers the violence we do to nature when we build a city. I can only refer to it, hoping by thus calling attention to it, you may have opportunity of reading it. Its perusal would repay for any trouble in looking it up, since it is by far the ablest article that has yet been written and published upon the subject of which it treats.

The air of a city would be greatly improved, if ventilators, in the form of huge chimneys connecting with the sewers, were constructed at suitable distances from each other on a line with the principal sewers of the city. London, I am informed, has adopted this method of carrying off the surplus sewer gas.

Certainly, by the adoption of this plan, not so much gas would be able to escape through traps into dwellings, nor would the surrounding atmosphere become so impregnated with gas, since the tall sewer chimney would convey it away, and the gas, being of greater specific levity than the atmospheric air, would rise and remain above that which we inhale.

If there be any reason why corner houses are less healthful than those situated midway in the block from the cross streets, it is in the fact, that at each crossing there are one or two inlets to receive surplus water drained from the gutters, and an equal number of outlets for surplus sewer gas to escape from, into the surrounding atmosphere. These open sewers are of course trapped, and are supposed to be secure, but they are, in dry seasons especially, sending forth poisonous exhalations, affecting first those who live in juxtaposition to them. The city authorities ought to make an appropriation for the payment of the services of a competent sanitarian, to whom should be delegated power sufficient to enforce such sanitary conditions, in the construction of dwellings and sewers, as he, in his wisdom, should regard as requisite and necessary for the public good.

Dr. Fergus, (*British Medical Journal*), at a meeting of the Birmingham Sanitary Conference, related the results of experiments, as to the value of water-trapping as a means of preventing sewer gas from entering dwelling-houses. The principle object he had in view, was to show that the few inches of water which, as a rule, are placed as a barrier between the sewers and the interior of houses, and which are dignified with the name of syphon-trap, afford, in reality, no protection against sewer poisoning.

Various gases were admitted without pressure into the tubes. The first experiment was carried out with ammonia, and it was found that in fifteen minutes, it had passed up the tube through the water, and had discharged the acid with which some litmus paper, suspended over the upper surface of the water, had been reddened. Very similar results were produced with other gases, quite irrespective of their being lighter or heavier than atmospheric air.

The results obtained by Dr. Fergus are interesting, as pointing out strongly, how misplaced is the confidence which the public have been led to repose in the traps with which nearly every house is provided, and how important it is, not only thoroughly to ventilate our house drains, but also to subject our soil pipes to periodical examination.

A disease, lately discussed abroad, and more recently in New York city, called pythogenic fever and pythogenic pneumonia, or sewer-gas fever and sewer-gas pneumonia, is a very protracted and fatal disease. It is not a new disease, but the discovery of the cause had not been made, I believe, until some time during the year 1873, or 1874. This is a disease that requires removal away from the exciting cause of it, provided the cause cannot be removed from the patient. This being conceded, how important it is to have early knowledge of the cause which has produced the malady, since safety of the patient entirely depends upon removal from it, and the necessity for much medication superceded.

Unless the ingathering of the multitude of children, who are victims of scarlet fever, be in accordance with, and part of, the Divine plan, by which sin in this world and punishment in the next are to be prevented, it is a consummation devoutly to be wished, that another Jenner may arise with his *preventive* method, that this fatal malady, which has been prowling about our city and country during the past two years, may as surely and effectually be prevented as is small-pox by vaccination.

Health gives strength to the individual, and imparts it to the state just in proportion to the degree and vigor of health. If we have a strong nation we must have strong men,—men of mental and men of physical strength,—and in order to be instrumental in rearing a stalwart race, our women must imitate the women of Sparta. Spartan women were educated to be mothers of a vigorous race.

Physical education of the citizen was the most remarkable feature of the legislation of Lycurgus; hence sanitary laws were encouraged and enforced upon the citizen.

The individual belonged to the state, was under the control of the state, and trained physically to become a hardy soldier. His discipline began at seven years of age, and marriage was allowed at the age of thirty years, when a new era and form of discipline began. We are not prepared to go as far as the Grecians in their desire to rear soldiers to strengthen the state, by allowing weak and feeble children to die because they weakened the nation, but we are willing that laws should be enacted to strengthen the constitution of children, such, for instance, as will prohibit unsuitable marital relations, the rental of untenantable houses, the conversion of private into public, or boarding houses, without provision against a bad atmosphere, which an aggregation of individuals engenders; in a word, we are in favor of all laws which will promote health, as well as give protection to property.

Sanitary science in a three-fold way increases the wealth of a nation.

First, it strives to prevent disease; secondly, it abridges the duration of, and mitigates the severity of disease; and thirdly, it saves human life; and since labor has a pecuniary value, the wealth of the state is enhanced by the saving of labor to the state.

Inestimable wealth would accrue to a nation were the laws of hygiene rigidly enforced. The aid of municipal, state and national authorities should be invoked in behalf of the grand work which is now carried on by the voluntary and unpaid labor of those, who, if less unselfish and more avaricious, would never labor to prevent that which gives them bread, and which, if prevented, dries up the sources of revenue.

Congress, while fitting out various scientific explorations, might very properly make liberal appropriations in behalf of a science that aims at the prevention of the causes of disease and the preservation of health.

